# BbGSD: Black-boned Sheep Genome SNP Database

**DOI:** 10.1093/database/baaf004

**Published:** 2025-01-28

**Authors:** Chunjuan He, Lichang Chen, Juntao Cao, Yuqing Zhong, Zhendong Gao, Weidong Deng, Jiajin Zhang

**Affiliations:** College of Big Data, Yunnan Agricultural University, 452 Fengyuan Road, Panlong District, Kunming, Yunnan 650201, China; College of Information, Lijiang Culture and Tourism College, 1 Yuquan Road, Gucheng District, Lijiang 674199, China; College of Big Data, Yunnan Agricultural University, 452 Fengyuan Road, Panlong District, Kunming, Yunnan 650201, China; College of Big Data, Yunnan Agricultural University, 452 Fengyuan Road, Panlong District, Kunming, Yunnan 650201, China; College of Animal Science and Technology, Yunnan Agricultural University, 452 Fengyuan Road, Panlong District, Kunming, Yunnan 650201, China; College of Animal Science and Technology, Yunnan Agricultural University, 452 Fengyuan Road, Panlong District, Kunming, Yunnan 650201, China; College of Animal Science and Technology, Yunnan Agricultural University, 452 Fengyuan Road, Panlong District, Kunming, Yunnan 650201, China; College of Big Data, Yunnan Agricultural University, 452 Fengyuan Road, Panlong District, Kunming, Yunnan 650201, China

## Abstract

Lanping black-boned (LPBB) sheep are a unique and rare ruminant species, characterized by black pigmentation in the skin and internal organs. Thus far, LPBB are the only known animal with heritable melanin characteristics besides the black-boned chicken, and the only mammal known to contain a large amount of melanin in the body. LPBB have therefore attracted substantial research attention, due to their potential contribution to medicine. However, long periods of grazing freely and crossbreeding with Lanping normal sheep (LPN) have diluted LPBB breeding resources, posing a challenge to the protection of species. To ensure the effective conservation and management of LPBB genetic resources, the construction of a large-scale database of genotypic information is therefore very important. To achieve this, we established the first LPBB-specific SNP database, named Black-boned Sheep Genome SNP Database (BbGSD, http://202.203.179.115:3838/oarsnpdb) using sheep genotype data (100 LPBB and 50 LPN) across 46 894 242 SNP sites. In this database, we implemented four main function modules: (i) the “LD heatmap” module, which uses a heatmap to enable the interactive visualization of pairwise linkage disequilibrium (LD) measurements between SNPs; (ii) the “SNP distribution” module, which allows users to interactively visualize tabular genotype data as heat maps; (iii) the “Phylogenetics” module which enables phylogenetic analysis to explore the evolutionary history or genetic relationships of the LPBB sheep; and the “Diversity” module, which can be used to calculate and display the nucleotide diversity among sheep populations in user-specified genomic regions. BbGSD is essential for accelerating studies on the functional genomics and screening of molecular markers of molecular-assisted breeding in black-boned sheep.

**Database URL**: http://202.203.179.115:3838/oarsnpdb

## Introduction

Sheep are key agricultural livestock not only for the production of meat and wool but are also an ideal animal model in biology and comparative genomics research [1]. China has some of the most extensive livestock and poultry genetic resources worldwide. However, these resources are seriously threatened due to their wide geographical distribution and uneven protection [2]. In general, the local livestock and poultry genetic resources in China are decreasing. Many local breeds are on the verge of extinction, and some have already become extinct [3]. Lanping black-boned (LPBB) sheep is a species of sheep that is found at high altitudes in the Yunnan Province of China. The difference between LPBB and other sheep is that the eye conjunctiva is brown, the skin of the elbow joints of the forelegs, hind limbs, hair roots and underarms is purple, the mouth and tongue are bright black, the gums are blue-green, and the anus is blue-purple. LPBB is the only mammal in the world with a large number of heritable melanin characteristics, similar to silky fowl. LPBB sheep have been recognized as a novel genetic resource by the Chinese Ministry of Agriculture [4], and were listed in the “National List of Animal and Poultry Genetic Resources,” “China Rare Animal Breeds List,” and “World Rare Animal Species List” [5]. To date, genes related to the mechanism of black pigmentation traits on LPBB have been studied such as tyrosinase, melanocortin 1 receptor [6], tyrosinase-related protein 1, tyrosinase-related protein 2 [7], and endothelin 3 [8]. Furthermore, Xiong *et al*. were the first to estimate the genetic diversity and genetic origin of LPBB by using genome-wide single-nucleotide polymorphism (SNP) data. This finding, through neighbor-joining (NJ) tree analysis and genetic differentiation studies, revealed that there are differences between the LPBB and Lanping normal sheep (LPN) populations, indicating that LPBB can be considered a specific and separate breed [9].

However, the majority of the LPBB sheep population is concentrated in the Lanping Bai Pumi Autonomous County (Lanping County) of the Nujiang Lisu Autonomous Prefecture (Nujiang Prefecture), a relatively remote area that experiences environmental extremes. Due to the small population and isolated habitat of LPBB, they likely have low genetic diversity and an increased incidence of inbreeding, which poses a threat to this unique and rare sheep breed [9]. Species with insufficient genetic diversity struggle to cope with environmental changes and adapt to evolving competitors and parasites [10]. Hence, studying the genetic diversity of LPBB sheep will undoubtedly play a vital role in its conservation, development, and utilization.

SNPs are defined as single base pair (bp) changes in a DNA sequence and are the most common form of sequence variation between individuals within a species [11]. They have become the most widely used class of genetic markers due to the richness of the genome and their adaptability for cost-effective high-throughput genotyping [12], and are widely used in population genetics studies (e.g. origin, evolution) and disease-associated gene research [13].

Thus far, rapid advances in sequencing technologies and other biotechnologies have greatly accelerated the generation of genomic big data. Although many bioinformatics tools have been exploited for the analysis of genomic data, the majority of tools rely on command-line environments or specific programming languages, making them challenging to use for researchers and breeders. To overcome this, the development of interactive biological web applications has been a major focus of bioinformatics research [14]. Numerous databases have been created to store and analyze SNPs of various species to make full use of genomic resources, such as DoGSD for dogs and wolves [15], ChickVD for chicken [16], and PigVar for pigs [17].

However, a database of LPBB SNPs has not yet been developed. As a result, the development of interactive web applications to deposit and analyze large-scale datasets will contribute to preserving LPBB genetic diversity and thus enable better conservation of these resources.

Here, we present the first LPBB-specific SNP database, Black-boned Sheep Genome SNP Database (BbGSD), which we constructed in R and implemented in Shiny [18, 19]. Analysis and visualization modules were constructed in BbGSD to visualize and explore 46 894 242 SNP sites among 150 sheep samples (100 LPBB and 50 LPN). In contrast to a simple “database,” BbGSD offers unique and powerful tools to help researchers’ breeders with in-depth analysis by providing fast and efficient access to large volumes of genomic variation data, without the need for programming skills. Furthermore, BbGSD provides novel insights for improved molecular breeding of LPBB sheep and future studies into heritable melanin characteristics.

## Materials and methods

### Data collection

We collected 443 adult LPBB and 298 adult LPN (local non-black sheep in Lanping) sheep from Lanping County. Each individual was over 1-year old and was verified to match the breed standard. A total of 100 unrelated LPBB sheep with particularly distinct melanin traits (50 males and 50 females) were then selected from 443 adult LPBB sheep, and 50 unrelated LPN sheep (25 males and 25 females) were selected from 298 LPN. Blood samples were collected from 100 LPBB and 50 LPN, and then preserved in freezers at −20°C prior to use. Genomic DNA was extracted from the blood using a TIANamp Genomic DNA Kit (TIANGEN, China), according to the manufacturer’s instructions. The integrity and purity of the DNA were determined using 1.5% agarose gel electrophoresis and testing using a NanoDrop 2000 (NanoDrop Technologies, USA). The DNA concentration was measured using a Qubit 2.0 (Life technologies, Japan). Aliquots where 1.5 μl samples of DNA were taken and library construction was performed according to the Truseg Nano DNA HT instructions (Illumina, USA). Ultrasonic waves were then used to fragment the DNA into 350 bp sections, after which end repair was performed, and A tails and DNA fragment connectors were added; finally, the PCR end-products were purified. Agilent 2100 (Agilent Technologies, USA) and real-time PCR (Thermo Fisher, USA) were used to conduct quality tests for fragment size and concentration on the constructed library. All the libraries were sequenced using the Illumina HiSeq 2500 (Illumina, USA); specifically, 150 bp of paired-end reads were generated, the insert size was ∼350 bp, and an average raw read sequence coverage of ∼9.3×.

### Data processing

The raw-sequencing data was 5369 GB of SNPs from 150 samples from previous studies, including 100 LPBB and 50 LPN [9]. The average sequencing quality Q30 was 91.14%, the average sequencing depth 9.3×, and the average Mapping Rate was 98.62% (Supplementary Table 1). The variant call format data for 100 LPBB and 50 LPN was extracted from the raw variant call format file. Using a Python script, the 0/0 genotypes of each population were removed, and the remaining sites represent the population’s SNP data. After comparing the SNPs of LPBB and LPN to identify the SNPs that differ from those of LPN, we counted the SNP allele frequencies and used a Python script to filter the SNPs with allele frequency ≥0.5, which are the SNPs that are unique to LPBB.

We further performed data quality control (QC) using Plink 1.9 [20, 21]. SNPs that did not pass the following criteria were removed: (i) minor allele frequency (MAF) > 0.01; (ii) missing genotype data < 0.10; (iii) individuals with missing genotype data < 10%; (iv) Hardy–Weinberg equilibrium *P*-value > .00001; and (v) located on autosomes. As a result, 46 894 242 SNP sites were obtained. The SNPs were annotated based on the sheep genome Oar_v4.1 reference genome [22] using SnpEff [23]. Moreover, we used a data structure implemented in R; specifically, the sheep genomic dataset was converted into an integer sparse matrix consisting of ‘0ʹ, ‘1ʹ, ‘2ʹ, and this dataset was then stored as an R data file [24]. We used a sparse matrix which can reduce the storage space and duration of computation.

### Database construction

BbGSD was implemented in R (version 4.2.2) using Shiny (version 1.8.0), run on a Linux server (Ubuntu OS version 20.04), and hosted on a Shiny server (version 1.5.17.973). Shiny was used to implement the user graphical interface and to process user input for customized data visualization. It is a powerful web framework that enables the construction of interactive web applications using R. Combining the computational power of R with the interactivity of the web [25] enabled not only data visualization and exploration but also the implementation of complex biological databases and web servers [26, 27].

The analysis features of BbGSD were implemented as independent R functions stored in R script files. The genotypes of 46 894 242 SNP sites among 150 sheep samples were stored as R data files in BbGSD, which can be flexibly used for genetic screening of specific genomic regions and SNP sites, and for generating a genotype table as intermediate data. The intermediate genotypic data were then stored in Random Access Memory (RAM) to enhance its capacity to be used in future analyses and visualization.

## Results and discussion

LPBB is a precious genetic resource of heritable melanin characteristics. Despite their conservation and potential medical importance, LPBB genetic diversity is low, predominantly due to harsh environmental conditions, such as high altitude (∼3000 m), strong radiation, and steep terrain. To help address this challenge, we utilized R/Shiny to build lightweight web applications for data storage, online analysis, and visualization of SNPs in LPBB and LPN. The interface of BbGSD was designed to be simple and user-friendly, which will be helpful for future studies on population genomics and screening of molecular markers for molecular-assisted breeding in the black-boned sheep.

**Figure 1.**
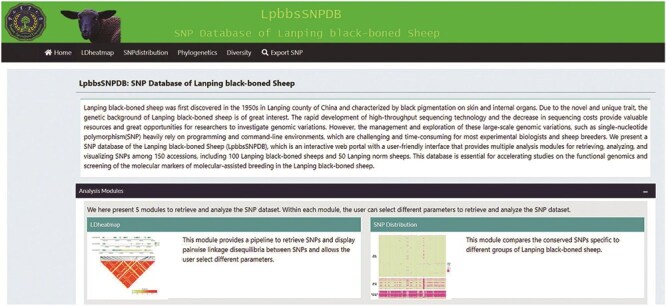
The graphical interface of BbGSD. The seven modules in the BbGSD application provide different functions.

The design of BbGSD includes seven modules: “Home,” “LD heatmap,” “SNP distribution,” “Phylogenetics,” “Diversity,” and “Export SNP,” to allow users to navigate massive genomic variation and perform lightweight analyses and visualization (Fig. 1). The “Home” module concisely introduces LPBB and each analysis module. The remaining functional modules provide versatile analysis and visualization opportunities, as discussed further. The graphical results and plain text files output by these modules can be downloaded in several formats for storage, publication, or further analysis by the user.

### LD heatmap

**Figure 2.**
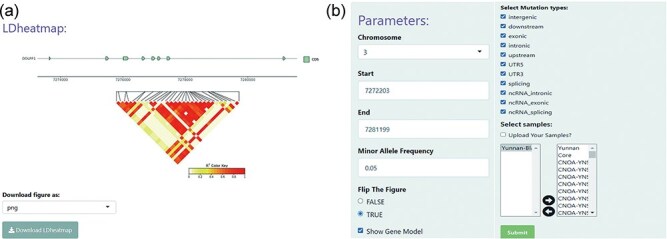
An example heat map generated using the LD heatmap module of BbGSD. (a) A heat map of patterns of LD, measured as *r*^2^. Each colored rectangle represents the squared correlation (*r*^2^) between a pair of SNPs. The structure of annotated gene models is displayed on top of the heat map. (b) User-interactive panel for filtering SNP site parameters.

The “LD heatmap” module uses a heatmap to enable interactive visualization of pairwise linkage disequilibrium (LD) measurements between SNPs. Understanding the patterns of association or LD among SNPs can aid investigations into population genetic processes. Specifically, the LD, measured in square coefficient of correlations (*r*^2^), between any pair of SNP sites for a user-specified genomic region were calculated and displayed as a heat map, using the R exhaustive LD heatmap [28]. The *r*^2^ is one of the most commonly used measures of LD. The degree of LD is represented by different rectangle color intensities. On top of the heat map, the structure of annotated gene models in the specified genomic region can be displayed (Fig. 2a). In addition, the module offers several simple and intuitive customization options, where the user can modify the MAF, SNP effects, and the sheep population being investigated. This customization enables the user to filter SNP site parameters (Fig. 2b).

### SNP distribution

**Figure 3.**
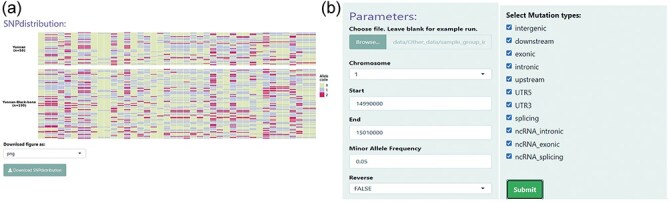
An example of the SNP distribution module. (a) Comparison of conserved SNPs specific to LPBB and LPN populations. (b) User-interactive panel for filtering SNP site parameters. Users can choose a specific genomic region and SNP effects and then upload their samples.

The “SNP distribution” module allows users to interactively visualize tabular genotype data as heat maps (Fig. 3a). This module greatly improves the capturing of group-specific haplotypes or genotype patterns on heat maps. Sample datasets should be uploaded by users to enable their own datasets to be explored. There is also a display option panel that contains SNP effect boxes and MAF select boxes that allow the user to filter different site parameters (Fig. 3b). By default, SNP sites and samples are displayed in columns and rows, respectively. SNPs that are homozygous (0) and heterozygous (1) for the reference allele or homozygous for the non-reference allele (2), are indicated by light-yellow, light blue, and red colors, respectively.

### Phylogenetics

**Figure 4.**
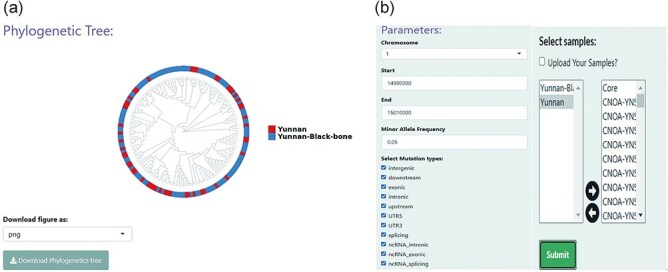
Phylogenetic analysis conducted using the “Phylogenetics” module in BbGSD. (a) NJ tree constructed based on SNPs around the MTURN gene, using the phylogenetic functionality of BbGSD. (b) User-interactive panel for filtering SNP site parameters.

The “Phylogenetics” module enables phylogenetic analysis, which helps the user explore the evolutionary history or genetic relationship of LPBB sheep (Fig. 4a). Calculation of genetic distances across sheep populations based on genotypes across all SNP sites were made for user-specified genomic regions (Fig. 4b). Based on genetic distances, the R package APE was then used to build a NJ tree [29]. An attractive feature of NJ tree analysis is that it enables the rapid evaluation of large volumes of data, i.e. for the exploration of genetic relationships between samples from specific regions in a relatively short time. Utilizing the ggtree package, an extension package to ggplot2, the NJ tree was visualized in a circular format [30]. The module can also be used to explore genetic structure. For instance, there was no significant separation between LPBB and LPN populations, which is consistent with previous studies (Fig. 4a) [1].

### Diversity

**Figure 5.**
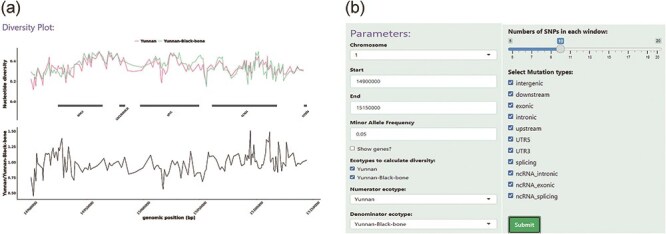
An example of the Diversity module. (**a**) The top panel displays the nucleotide diversity between LPBB and LPN sheep, indicated by different colors. The bottom panel shows the division of nucleotide diversity in LPBB and LPN. (b) An option panel contains SNP sliders and SNP effects select boxes that allow the user to restrict certain SNP sites.

The “Diversity” module allows the user to calculate and display the nucleotide diversity among sheep populations in user-specified genomic regions (Fig. 5a). The degree of polymorphism within a population is measured by nucleotide diversity. A unified panel was built, which enables flexible filtering of different parameters to restrict the SNP sites used in calculations and visualization (Fig. 5b). User-specified genomic regions were split into non-overlapping genomic regions, each of which contained 10 SNP sites (default value). The R package pegas was used to calculate the nucleotide diversity of each genomic region belonging to a specific sheep population; this information was then shown as a line chart using the R package ggplot2 [31, 32]. The user can select whether the gene model is demonstrated in the specified genomic region.

### Export SNPs

The “Export SNPs” module allows users to retrieve and freely download all built-in data, including gene data, SNP data, and accession data, from the BbGSD database. Notably, detailed information and geographical distributions of the sheep, as selected by the user, are visible in tabular form, and are available for download in several file formats (txt, xlsx, and csv).

In the present version of BbGSD, only SNPs are included. Future integration of rich new genomic data with transcriptomics, genetic mapping, genetic markers, trait loci, and phenotypic data may provide more detailed insights into the melanic traits and genetic diversity of LPBB sheep. Essentially, BbGSD will be continually updated whenever new LPBB genetic data are released. Furthermore, new relevant data mining and analysis modules will be constantly developed. Taken together, the overall goal of BbGSD is to provide long-term storage for LPBB omics data and to offer visualization tools for data mining and analysis.

## Conclusions

LPBB is a unique and rare genetic resource. BbGSD, as an LPBB-specific SNP database, enables genomic selection and conservation of LPBB genetic resources by researchers and breeders. Herein, several popular genetic analysis modules in BbGSD were implemented with a graphical interface, which provides the user with the flexibility to analyze specific genomic regions, and thus explore SNP data interactively. In particular, almost all analysis module calculations can be performed in real-time, with the results displayed immediately on the main panel. Additionally, a range of different input parameters can be quickly tested through the parameter panel, enabling rapid and efficient data analysis. Moreover, BbGSD offers data visualization, searching, and freely available downloads for all users. All data in BbGSD are publicly available without any restrictions.

Researchers and breeders worldwide are welcome to submit comments and suggestions. Through continuous updates, we believe BbGSD can provide a foundation for future sustainable genetic improvement and conservation programs for LPBB sheep, a precious genetic resource.

## Supplementary Material

baaf004_Supp

## Data Availability

Database homepage: http://202.203.179.115:3838/oarsnpdb. The database is freely available without restrictions for use in academic research.
