# Supplementary material for: BbGSD: Black-boned Sheep Genome SNP Database
Source: Database (Oxford). 2025 Jan 28;2025:baaf004. doi: 10.1093/database/baaf004 (PMC11774206; doi:10.1093/database/baaf004)
Supplement: baaf004_Supp [file baaf004_supp.zip › suppl_data/Appendix.docx]

# Appendix

Supplementary table 1: Sample information involved in this study (100 LPBB and 50 LPN)

| Sample number | Breed | Rawbase（Gp） | Q30(%) | Mapping rate(%) | Depth**（**×**）** |
| --- | --- | --- | --- | --- | --- |
| K138 | Lanping black-boned sheep | 34.2 | 91.01 | 99.08 | 9.1592 |
| DK123 | Lanping black-boned sheep | 30.9 | 86.06 | 98.98 | 9.5819 |
| DK124 | Lanping black-boned sheep | 33.1 | 86.83 | 98.90 | 8.9364 |
| DK130 | Lanping black-boned sheep | 34.6 | 86.29 | 96.31 | 8.8681 |
| DK135 | Lanping black-boned sheep | 29.8 | 85.69 | 98.75 | 9.0274 |
| DK137 | Lanping black-boned sheep | 37.1 | 86.26 | 95.62 | 8.7881 |
| Sample number | Breed | Rawbase（Gp） | Q30(%) | Mapping rate(%) | Depth**（**×**）** |
| DK139 | Lanping black-boned sheep | 36.4 | 87.92 | 98.68 | 9.3136 |
| DK151 | Lanping black-boned sheep | 30.4 | 87.65 | 98.61 | 9.47 |
| DK152 | Lanping black-boned sheep | 30.2 | 86.85 | 97.22 | 9.8365 |
| DK161 | Lanping black-boned sheep | 36.9 | 87.76 | 99.06 | 9.4043 |
| DK165 | Lanping black-boned sheep | 28.1 | 85.86 | 97.98 | 9.1963 |
| DG51 | Lanping black-boned sheep | 33.7 | 93.18 | 98.71 | 9.4837 |
| DG59 | Lanping black-boned | 33.2 | 92.69 | 98.88 | 8.8612 |
| DG61 | Lanping black-boned | 35.9 | 90.38 | 96.89 | 9.0975 |
| DG62 | Lanping black-boned sheep | 33.9 | 90.56 | 97.67 | 9.0566 |
| DG64 | Lanping black-boned sheep | 33.7 | 91.19 | 93.64 | 8.9566 |
| DG66 | Lanping black-boned sheep | 33.9 | 90.62 | 98.95 | 9.4759 |
| DG67 | Lanping black-boned sheep | 62.0 | 87.34 | 98.01 | 8.8623 |
| DG75 | Lanping black-boned sheep | 33.1 | 90.44 | 99.28 | 8.4761 |
| DG82 | Lanping black-boned sheep | 34.9 | 93.09 | 96.76 | 8.6456 |
| DG91 | Lanping black-boned sheep | 34.6 | 91.64 | 99.49 | 8.4175 |
| DG94 | Lanping black-boned sheep | 35.6 | 92.18 | 94.62 | 8.5056 |
| DG95 | Lanping black-boned sheep | 34.7 | 92.28 | 99.31 | 8.7278 |
| DG96 | Lanping black-boned sheep | 32.0 | 92.64 | 99.13 | 8.5574 |
| DG97 | Lanping black-boned sheep | 33.7 | 91.53 | 99.48 | 9.0459 |
| DG98 | Lanping black-boned sheep | 32.0 | 91.76 | 98.36 | 11.2502 |
| DG112 | Lanping black-boned sheep | 32.8 | 91.26 | 99.27 | 9.851 |
| DG117 | Lanping black-boned sheep | 53.6 | 85.02 | 99.35 | 9.1669 |
| DG118 | Lanping black-boned sheep | 34.0 | 92.06 | 99.41 | 8.8872 |
| DG119 | Lanping black-boned sheep | 31.5 | 92.41 | 99.13 | 9.6033 |
| DG120 | Lanping black-boned sheep | 34.0 | 91.76 | 96.98 | 8.6879 |
| DG123 | Lanping black-boned sheep | 64.7 | 86.2 | 99.68 | 9.593 |
| DG143 | Lanping black-boned sheep | 34.5 | 91.17 | 97.57 | 9.8916 |
| DG152 | Lanping black-boned sheep | 35.8 | 91.61 | 95.71 | 8.2703 |
| DG158 | Lanping black-boned sheep | 33.3 | 91.12 | 97.66 | 10.4706 |
| DG173 | Lanping black-boned sheep | 34.3 | 93.67 | 97.41 | 10.9952 |
| DG177 | Lanping black-boned sheep | 34.7 | 92.4 | 96.82 | 9.0961 |
|  |  |  |  |  |  |
| Sample number | Breed | Rawbase（Gp） | Q30(%) | Mapping rate(%) | Depth**（**×**）** |
| DG179 | Lanping black-boned sheep | 36.4 | 93.96 | 99.54 | 9.1034 |
| DG192 | Lanping black-boned sheep | 34.3 | 94.05 | 96.95 | 11.0745 |
| DG200 | Lanping black-boned sheep | 34.5 | 93.4 | 96.67 | 8.0829 |
| DG201 | Lanping black-boned sheep | 35.1 | 93.9 | 99.06 | 8.8901 |
| DG213 | Lanping black-boned sheep | 32.6 | 92.4 | 99.33 | 8.9985 |
| DG219 | Lanping black-boned sheep | 34.5 | 92.71 | 98.90 | 8.9826 |
| DG222 | Lanping black-boned sheep | 34.1 | 92.44 | 99.41 | 8.7698 |
| DG228 | Lanping black-boned sheep | 66.1 | 86.18 | 98.74 | 9.5211 |
| DG229 | Lanping black-boned sheep | 35.0 | 92.69 | 99.33 | 8.6656 |
| DG230 | Lanping black-boned sheep | 33.2 | 92.34 | 98.80 | 9.1838 |
| DG237 | Lanping black-boned sheep | 31.7 | 91.47 | 98.75 | 9.467 |
| DG238 | Lanping black-boned sheep | 32.4 | 91.53 | 99.34 | 8.6614 |
| XL1 | Lanping black-boned sheep | 32.0 | 92.72 | 98.89 | 9.4214 |
| XL2 | Lanping black-boned sheep | 32.9 | 91.75 | 94.97 | 8.6086 |
| XL3 | Lanping black-boned sheep | 34.4 | 92.64 | 98.25 | 9.4972 |
| XL6 | Lanping black-boned sheep | 34.8 | 93.04 | 98.34 | 9.8915 |
| XL10 | Lanping black-boned sheep | 34.6 | 92.59 | 98.21 | 9.1108 |
| XL20 | Lanping black-boned sheep | 34.1 | 93.07 | 97.39 | 9.284 |
| XL21 | Lanping black-boned sheep | 33.5 | 92.83 | 99.47 | 8.6147 |
| XL22 | Lanping black-boned sheep | 33.4 | 92.6 | 97.46 | 9.478 |
| XL36 | Lanping black-boned sheep | 35.7 | 93.01 | 98.90 | 8.8864 |
| XL37 | Lanping black-boned sheep | 32.9 | 92.46 | 98.62 | 9.5829 |
| XL38 | Lanping black-boned sheep | 42.2 | 92.72 | 98.91 | 9.946 |
| XL39 | Lanping black-boned sheep | 34.3 | 92.89 | 98.98 | 9.7944 |
| XL40 | Lanping black-boned sheep | 34.0 | 91.82 | 99.06 | 9.4739 |
| XL41 | Lanping black-boned sheep | 33.8 | 92.34 | 98.91 | 9.4714 |
| XL42 | Lanping black-boned sheep | 33.9 | 91.99 | 99.07 | 9.7373 |
| XL43 | Lanping black-boned sheep | 34.0 | 92.27 | 99.12 | 9.1055 |
| XL44 | Lanping black-boned sheep | 34.4 | 92.56 | 96.22 | 8.6823 |
| XL45 | Lanping black-boned sheep | 38.8 | 93.09 | 99.58 | 9.0957 |
| XL54 | Lanping black-boned sheep | 41.1 | 93.42 | 98.38 | 8.9026 |
|  |  |  |  |  |  |
| Sample number | Breed | Rawbase（Gp） | Q30(%) | Mapping rate(%) | Depth**（**×**）** |
| XL55 | Lanping black-boned sheep | 34.8 | 93.26 | 99.46 | 8.8018 |
| XL57 | Lanping black-boned sheep | 36.2 | 93.13 | 99.60 | 8.9116 |
| XL60 | Lanping black-boned sheep | 33.9 | 92.62 | 99.20 | 9.0591 |
| XL65 | Lanping black-boned sheep | 34.2 | 93.06 | 98.99 | 8.6984 |
| XL75 | Lanping black-boned sheep | 33.5 | 92.66 | 98.96 | 8.962 |
| XL80 | Lanping black-boned sheep | 33.4 | 93.06 | 99.54 | 9.0368 |
| XL87 | Lanping black-boned sheep | 33.4 | 92.72 | 99.37 | 9.1394 |
| XL89 | Lanping black-boned sheep | 36.2 | 93.11 | 98.97 | 9.3071 |
| XL90 | Lanping black-boned sheep | 34.7 | 92.91 | 99.56 | 9.6691 |
| XL91 | Lanping black-boned sheep | 34.2 | 92.58 | 99.39 | 8.7627 |
| XL92 | Lanping black-boned sheep | 34.5 | 91.59 | 99.45 | 11.025 |
| XL96 | Lanping black-boned sheep | 33.5 | 92.5 | 97.78 | 9.12 |
| XL101 | Lanping black-boned sheep | 34.5 | 92.85 | 98.13 | 9.1223 |
| XL111 | Lanping black-boned sheep | 35.3 | 93.18 | 99.45 | 9.0787 |
| XL118 | Lanping black-boned sheep | 35.0 | 92.53 | 98.15 | 9.0714 |
| XL126 | Lanping black-boned sheep | 35.5 | 93.03 | 99.04 | 9.2105 |
| XL144 | Lanping black-boned sheep | 87.3 | 93.19 | 99.55 | 9.4819 |
| XL161 | Lanping black-boned sheep | 35.7 | 93.28 | 99.54 | 10.4109 |
| XL177 | Lanping black-boned sheep | 34.0 | 92.85 | 99.24 | 10.5532 |
| XL184 | Lanping black-boned sheep | 33.4 | 93.15 | 99.55 | 9.1758 |
| XL206 | Lanping black-boned sheep | 32.0 | 92.72 | 99.39 | 9.6194 |
| K90 | Lanping black-boned sheep | 33.7 | 90.77 | 98.95 | 9.0829 |
| DG304 | Lanping black-boned sheep | 34.0 | 92.23 | 99.46 | 9.0865 |
| DJ3 | Lanping black-boned sheep | 58.5 | 85.29 | 99.35 | 9.3114 |
| DJ6 | Lanping black-boned sheep | 34.0 | 91.39 | 99.42 | 9.0595 |
| DK26 | Lanping black-boned sheep | 32.4 | 90.92 | 99.57 | 8.8962 |
| DK90 | Lanping black-boned sheep | 55.4 | 88.83 | 99.53 | 8.9225 |
| DK92 | Lanping black-boned sheep | 32.7 | 91.67 | 99.31 | 9.6601 |
| DK172 | Lanping black-boned sheep | 33.9 | 91.47 | 99.14 | 8.9948 |
| DK173 | Lanping black-boned sheep | 33.9 | 91.77 | 99.33 | 9.081 |
| DK176 | Lanping black-boned sheep | 34.7 | 92.21 | 99.43 | 9.1966 |
|  |  |  |  |  |  |
| Sample number | Breed | Rawbase（Gp） | Q30(%) | Mapping rate(%) | Depth**（**×**）** |
| DK178 | Lanping black-boned sheep | 61.9 | 86.36 | 99.10 | 9.5203 |
| DK83 | Lanping normal sheep | 32.4 | 87.74 | 98.30 | 8.9134 |
| DK169 | Lanping normal sheep | 33.5 | 91.45 | 98.52 | 9.2828 |
| DG249 | Lanping normal sheep | 33.6 | 92.18 | 99.25 | 9.7721 |
| DG250 | Lanping normal sheep | 34.1 | 91.99 | 99.40 | 8.6052 |
| DG267 | Lanping normal sheep | 34.5 | 92.19 | 98.58 | 8.9377 |
| DG270 | Lanping normal sheep | 32.3 | 89.5 | 98.56 | 8.8236 |
| DG305 | Lanping normal sheep | 33.8 | 92.34 | 99.28 | 9.2477 |
| DG306 | Lanping normal sheep | 33.7 | 90.37 | 99.18 | 8.7548 |
| DG343 | Lanping normal sheep | 31.8 | 91.84 | 98.91 | 9.354 |
| DG345 | Lanping normal sheep | 32.2 | 93.23 | 99.41 | 9.5222 |
| DK179 | Lanping normal sheep | 35.2 | 89.79 | 99.24 | 9.3297 |
| DK180 | Lanping normal sheep | 32.5 | 92.37 | 98.87 | 8.5562 |
| DK181 | Lanping normal sheep | 33.7 | 89.47 | 98.78 | 9.4294 |
| DK182 | Lanping normal sheep | 34.0 | 89.77 | 99.03 | 9.2131 |
| DK187 | Lanping normal sheep | 33.5 | 89.63 | 99.23 | 8.688 |
| DK188 | Lanping normal sheep | 34.1 | 90.08 | 99.42 | 8.8678 |
| DK189 | Lanping normal sheep | 31.0 | 92.72 | 99.13 | 8.8436 |
| DK190 | Lanping normal sheep | 30.9 | 92.94 | 94.46 | 8.523 |
| DK191 | Lanping normal sheep | 34.7 | 89.53 | 99.06 | 9.8073 |
| DK192 | Lanping normal sheep | 33.8 | 90.8 | 98.97 | 9.5189 |
| PT19 | Lanping normal sheep | 34.9 | 91.28 | 99.25 | 9.3351 |
| PT20 | Lanping normal sheep | 34.7 | 90.5 | 99.45 | 8.6404 |
| PT21 | Lanping normal sheep | 33.6 | 90.23 | 98.48 | 9.5466 |
| PT22 | Lanping normal sheep | 33.7 | 93.56 | 98.76 | 9.2114 |
| PT56 | Lanping normal sheep | 33.0 | 93.06 | 99.57 | 9.9114 |
| PT57 | Lanping normal sheep | 33.7 | 93.13 | 99.59 | 12.0204 |
| PT60 | Lanping normal sheep | 34.2 | 93.24 | 98.96 | 9.8043 |
| PT91 | Lanping normal sheep | 33.0 | 92.89 | 99.40 | 10.1253 |
| PT97 | Lanping normal sheep | 33.5 | 92.97 | 97.09 | 8.4866 |
| PT118 | Lanping normal sheep | 33.6 | 92.04 | 99.42 | 10.9057 |
|  |  |  |  |  |  |
| Sample number | Breed | Rawbase（Gp） | Q30(%) | Mapping rate(%) | Depth**（**×**）** |
| PT132 | Lanping normal sheep | 33.0 | 93.14 | 99.35 | 13.6368 |
| PT139 | Lanping normal sheep | 34.6 | 93.9 | 99.06 | 8.2696 |
| PT150 | Lanping normal sheep | 57.8 | 84.9 | 99.59 | 10.0589 |
| PT152 | Lanping normal sheep | 33.0 | 93 | 99.55 | 13.4607 |
| PT154 | Lanping normal sheep | 35.1 | 92.62 | 97.68 | 9.5179 |
| PT187 | Lanping normal sheep | 34.6 | 93.14 | 95.39 | 8.7248 |
| PT188 | Lanping normal sheep | 33.6 | 92.04 | 99.07 | 9.6896 |
| PT192 | Lanping normal sheep | 33.8 | 93.72 | 99.52 | 9.009 |
| PT196 | Lanping normal sheep | 32.4 | 92.51 | 93.37 | 8.9133 |
| PT200 | Lanping normal sheep | 33.6 | 93.26 | 98.83 | 9.3269 |
| D161 | Lanping normal sheep | 32.3 | 87.08 | 99.15 | 9.2113 |
| D187 | Lanping normal sheep | 38.8 | 86.97 | 99.48 | 9.2534 |
| D188 | Lanping normal sheep | 32.5 | 87.02 | 99.18 | 8.7037 |
| D252 | Lanping normal sheep | 33.2 | 87.01 | 98.13 | 8.7754 |
| DK109 | Lanping normal sheep | 29.0 | 86.64 | 99.15 | 8.7007 |
| DK79 | Lanping normal sheep | 37.5 | 86.73 | 98.93 | 9.3359 |
| DK84 | Lanping normal sheep | 47.5 | 86.11 | 98.97 | 8.8615 |
| DK85 | Lanping normal sheep | 27.5 | 87.17 | 98.64 | 9.3966 |
| DK86 | Lanping normal sheep | 34.3 | 86.92 | 99.19 | 9.6033 |
| DK89 | Lanping normal sheep | 45.2 | 87.51 | 99.33 | 8.597 |
| Average |  | 35.8 | 91.14 | 98.62 | 9.3 |
